# SUDOSCAN, an Innovative, Simple and Non-Invasive Medical Device for Assessing Sudomotor Function

**DOI:** 10.3390/s22197571

**Published:** 2022-10-06

**Authors:** Dana Elena Gavan, Alexandru Gavan, Cosmina Ioana Bondor, Bogdan Florea, Frank Lee Bowling, Georgeta Victoria Inceu, Liora Colobatiu

**Affiliations:** 1 Clinic of Podiatry, 10 Iuliu Moldovan Street, 400348 Cluj-Napoca, Romania; 2 Department of Medical Devices, Faculty of Pharmacy, Iuliu Hatieganu University of Medicine and Pharmacy, 4 Louis Pasteur Street, 400349 Cluj-Napoca, Romania; 3 Department of Medical Informatics and Biostatistics, Faculty of Medicine, Iuliu Hatieganu University of Medicine and Pharmacy, 6 Louis Pasteur Street, 400349 Cluj-Napoca, Romania; 4 Faculty of Medicine, University of Manchester, Oxford Road, Manchester M13 9PL, UK; 5 Department of Vascular Surgery and Reconstructive Microsurgery, “Victor Babes” University of Medicine and Pharmacy, 2 Eftimie Murgu Square, 300041 Timisoara, Romania; 6 Department of Diabetes, Nutrition and Metabolical Diseases, Faculty of Medicine, Iuliu Hatieganu University of Medicine and Pharmacy, 2–4 Clinicilor Street, 400006 Cluj-Napoca, Romania

**Keywords:** cardiac autonomic neuropathy, diabetes mellitus, medical device, sudomotor function, SUDOSCAN

## Abstract

Diabetic autonomic neuropathy is probably the most undiagnosed but serious complication of diabetes. The main objectives were to assess the prevalence of peripheral and autonomic neuropathy in a population of diabetic patients, analyze it in a real-life outpatient unit scenario and determine the feasibility of performing SUDOSCAN tests together with widely used tests for neuropathy. A total of 33 patients were included in the study. Different scoring systems (the Toronto Clinical Neuropathy Score—TCNS; the Neuropathy Disability Score—NDS; and the Neuropathy Symptom Score—NSS) were applied to record diabetic neuropathy (DN), while the SUDOSCAN medical device was used to assess sudomotor function, detect diabetic autonomic neuropathy and screen for cardiac autonomic neuropathy (CAN). Fifteen (45.5%) patients had sudomotor dysfunction. The SUDOSCAN CAN risk score was positively correlated with the hands’ electrochemical sweat conductance (ESC), diastolic blood pressure (DBP), the level of the glycated hemoglobin, as well as with the TCNS, NDS and NSS. Performing SUDOSCAN tests together with other tests for DN proved to be a feasible approach that could be used in daily clinical practice in order to screen for DN, as well as for the early screening of CAN, before more complex and time-consuming tests.

## 1. Introduction

Diabetes mellitus is one of the most common metabolic disorders worldwide, resulting from a defect in insulin secretion, insulin action or both [1,2]. The latest literature on the matter suggests that diabetes is one of the most rapidly increasing diseases of our era. This health condition has been dramatically growing, from more than 275 million cases in 2010 to an estimated 537 million in 2021. Moreover, the number of diabetic patients is expected to continue rising to an estimated 643 million by 2030 and 783 million by 2045 [3]. At the present moment, chronic microvascular (retinopathy, neuropathy and nephropathy) and macrovascular (myocardial infarction, cerebrovascular diseases and peripheral arterial disease) complications remain important problems. They are associated with a significant burden on a patients’ morbidity and quality of life where the health care costs are staggering [4,5]. Diabetic neuropathy (DN) is typically described as the “presence of symptoms and/or signs of peripheral nerve dysfunction in people with diabetes, after the exclusion of other causes” [6]. DN represents the primary global neuropathy and is one of the most predominant diabetic complications, affecting up to 60% of patients over their lifetime [7,8].

Diabetic neuropathy can manifest in various forms, among which the most common are distal polyneuropathy and cardiac autonomic neuropathy (CAN) [2]. Probably the most undiagnosed DN is the autonomic neuropathy, which is a serious and often underestimated complication of diabetes [8,9]. 

The detection of DN in general and of autonomic neuropathy in particular, especially in their early stages, is considered to be extremely important in complication management [6]. Developing innovative, non-invasive, simple and quick screening tests for the diagnosis of peripheral and autonomic neuropathy could prove to be extremely clinically useful.

However, at present, there are limited tools to detect DN and CAN in their early stages; some tests are difficult to perform or require specially trained personnel [10,11]. 

The sudomotor function has been included by the American Diabetes Association (ADA) in the early diagnosis of autonomic neuropathy in diabetic patients. Sudomotor function is largely regulated by small, unmyelinated cholinergic sympathetic C-fibers that also regulate cardiovascular autonomic function, which may be affected earlier in patients experiencing DN [12].

Currently, one commonly used test of sudomotor function is quantitative sudomotor axon reflex testing (QSART), but performing it imposes some technical challenges, as it demands the careful control of testing conditions and patient positioning to ensure reliability [13]. 

SUDOSCAN represents an innovative, simple and non-invasive medical device which enables the assessment of sudomotor function by measuring the electrochemical sweat conductance (ESC). The apparatus can also provide a CAN risk score to forecast the possible risk of developing CAN.

The main objectives were to assess the prevalence of peripheral and autonomic neuropathy in a population of diabetic patients, analyze it in a real-life outpatient unit scenario and determine the feasibility of performing SUDOSCAN tests together with consecrated tests for neuropathy. Furthermore, we intended to investigate the correlation between SUDOSCAN results and other measures of DN. 

## 2. Materials and Methods

### 2.1. Patients

The study was conducted in a specialized podiatry clinic in Cluj-Napoca, Romania, from January to March 2017. Host institution ethical approval had previously been obtained for the study, released under Nr. 01/06.03-28.04.2017, and all subjects signed written informed consent.

Type 2 diabetes patients with or without symptoms of diabetic neuropathy consented to the study. Exclusion criteria included: pregnancy or nursing, amputation, implanted medical devices, actively bleeding lacerations to the arms and legs and a history of seizures or epilepsy.

### 2.2. Methods

All patients were examined by a physician. Age, sex, weight, height, body mass index (BMI), medical history of diabetes, resting systolic and diastolic blood pressure, antihypertensive treatment, existing diabetes complications (retinopathy, nephropathy or others) and the value of the glycated hemoglobin were documented. The blood pressure was recorded after 5 min of rest in a supine position. The glycated hemoglobin was determined using an automatic analyzer (DCA Vantage TM, Siemens, Erlangen, Germany) [14,15].

For every patient, a detailed foot assessment was performed as described by Boulton et al. [16], with the feet being carefully examined for dryness, fissures, calluses, ulceration, amputations, gangrene, infection/inflammation, the presence of foot pulses and Charcot arthropathy. The hands of the patients were also investigated for dryness, calluses, deformations, amputations or any skin conditions affecting their palms, such as atopic dermatitis.

#### 2.2.1. Neuropathy Assessment

The Toronto Clinical Neuropathy Score (TCNS), the Neuropathy Disability Score (NDS) and the Neuropathy Symptom Score (NSS) were used to evaluate DN. The TCNS was first adopted by a research group in Toronto for the screening of diabetic peripheral neuropathy (DPN) [2]. It consists of three parts: symptom scores, reflex scores and sensory test scores. In this study, the following criteria for the classification of DPN were used: 0 to 5 points, without DPN; 6 to 8 points, mild DPN; 9 to 11 points, moderate DPN; and 12 to 19 points, severe DPN [17]. 

The NDS includes the ankle reflex, vibration, pinprick and temperature (cold tuning fork) sensation at both sides of the great toes, with a maximum score of 10 points. Those with NDSs of 6 points or more were considered abnormal [2,18]. 

The NSS consists of muscle weakness, sensory disturbances and autonomic symptoms and can be further divided into 17 items. Items that are answered as negative/absent are scored 0, with presence being scored as 1 point. Patients with NSSs of 3–4 points were considered to present mild symptoms, 5–6 points-moderate symptoms, while 7–10 points indicated severe symptoms [2].

#### 2.2.2. Assessment of Sudomotor Function

In order to minimize any possible interference and to obtain clean sudomotor function assessments, patients were instructed to avoid applying any ointments or lotions.

**Figure 1:**
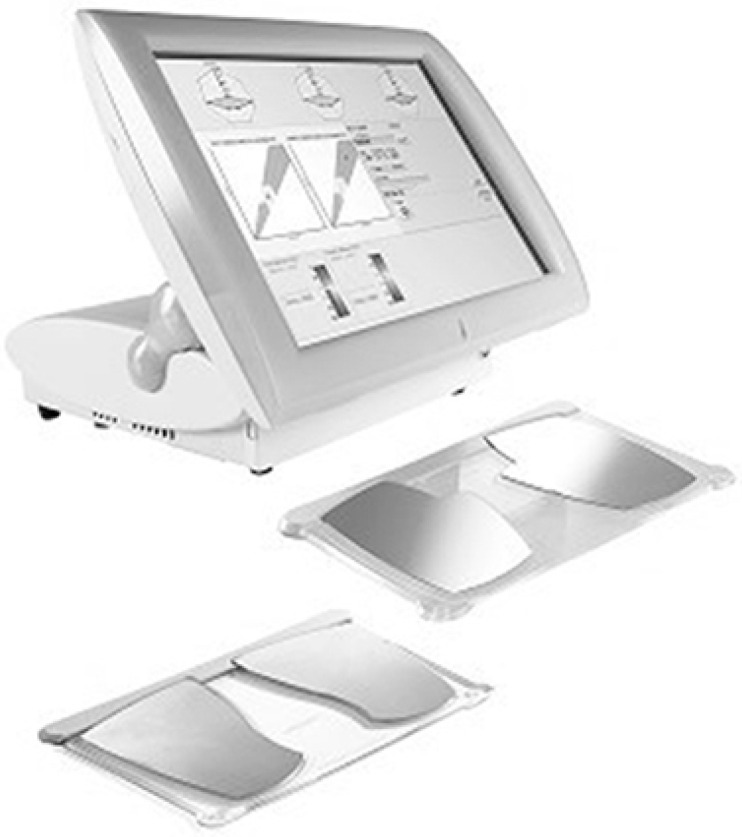
SUDOSCAN medical device—general presentation.

SUDOSCAN (Impeto Medical; Paris, France) was used to assess sudomotor function based on reverse iontophoresis and chronoamperometry (Figure 1). The method has been validated in several clinical studies [13,19,20,21,22]. 

The device consists of two sets of large surface stainless steel electrodes: one set for the palms and one for the soles. Both sets were connected on a desktop computer. The patients were instructed to place both hands and feet simultaneously on the designated electrodes. Scanning took 2–3 min to complete and was completely painless. A low direct current voltage, ranging from 1 to 4 V, was incrementally applied to the electrodes, with the left and right electrodes serving alternatively as cathode and anode. At voltages ≤10 V, the thick stratum corneum is electrically insulating; however, due to their cellular bilayer, the sweat glands can transmit electrically charged ions to the skin surface and further to the electrodes (reverse iontophoresis).

The current of sweat chloride ions generated in response to the incremental voltage applied can be quantified and reflects the function of the sweat gland, and hence its C-fiber innervation. Finally, this chloride ion current is measured in microSiemens (μS) and reported as ESC, expressed as the ratio of the current generated and the constant DC stimulus [4,23]. According to the measured values of the ESC, patients were classified into the following categories: without autonomic neuropathy (hands and feet ESC > 60 µS); with possible autonomic neuropathy (hands and feet ESC ≥ 40 µS and ≤60 µS); and with advanced autonomic neuropathy (hands or feet ESC < 40 µS). Threshold values were defined in previous research [1,19,24]. The device also has built-in algorithms which calculate ESC as a function of age, height, weight and the glycated hemoglobin level in order to generate a score value estimating the current CAN risk (SUDOSCAN-CAN risk score) [25]. The SUDOSCAN CAN risk score was classified as follows: no (or low) risk for CAN (SUDOSCAN-CAN risk score < 25%); moderate risk (SUDOSCAN-CAN risk score ≥ 25 and <50%); and high risk (SUDOSCAN-CAN risk score ≥ 50%).

#### 2.2.3. Statistical Analysis

Data were presented according to the type of variables studied: quantitative variables by synthetic descriptive indicators and qualitative variables by absolute and relative frequencies. The correlation between two quantitative or categorical qualitative variables was determined using Pearson or Spearman correlation coefficients. Chi-square or Fisher’s exact tests were used for the comparison of qualitative variables, while Student’s t or Mann–Whitney tests were applied in the case of quantitative or ordinal qualitative variables, respectively.

In order to control the influence of some parameters on the risk of autonomic neuropathy, a multivariate linear regression analysis was performed. 

A *p*-value < 0.05 was regarded as statistically significant. The statistical analysis was performed using the SPSS application (IBM, New York, NY, USA) and Microsoft Excel 2016 (Microsoft, Washington, DC, USA).

## 3. Results

A total of 38 patients were identified through screening, but were 5 excluded due to foot ulceration. Thirty-three patients therefore consented. The demographic and clinical characteristics of the patients are presented in Table 1.

The mean age of the patients was 64.6 ± 16.0 years, and 18 (54.5%) were men. All patients were diagnosed with type 2 diabetes (mean diabetes duration 12.9 ± 9.3 years). Among the 33 patients, 1 (3.0%) previously had an amputation (osteotomy) and 2 (6.1%) had Charcot foot. Moreover, 10 (30.3%) had retinopathy, 8 (24.2%) arteriopathy and 2 (6.1%) had nephropathy. The mean systolic blood pressure (SBP) was 149.6 ± 23.1 mmHg, while the mean diastolic blood pressure (DBP) was 82.7 ± 9.7 mmHg. Twenty-six patients (78.8%) were receiving antihypertensive treatment, but out of these, 18 (69.2%) had an SBP > 140 mmHg, while 10 (38.5%) of them had a DBP > 90 mmHg. Among the other seven patients that did not receive antihypertensive treatment, five (71.4%) had SBP > 140 mmHg and the other five (71.4%) had DBP > 90 mmHg. The mean level of glycated hemoglobin was 7.5 ± 1.5%.

According to the assessment of the sudomotor function performed by SUDOSCAN, 15 (45.5%) patients had sudomotor dysfunction. Out of these, seven (21.2%) possibly had autonomic neuropathy (hands and feet ESC ≥ 40 µS, but ≤60 µS), while eight (24.2%) had advanced autonomic neuropathy (hands or feet ESC < 40 µS).

**Figure 2:**
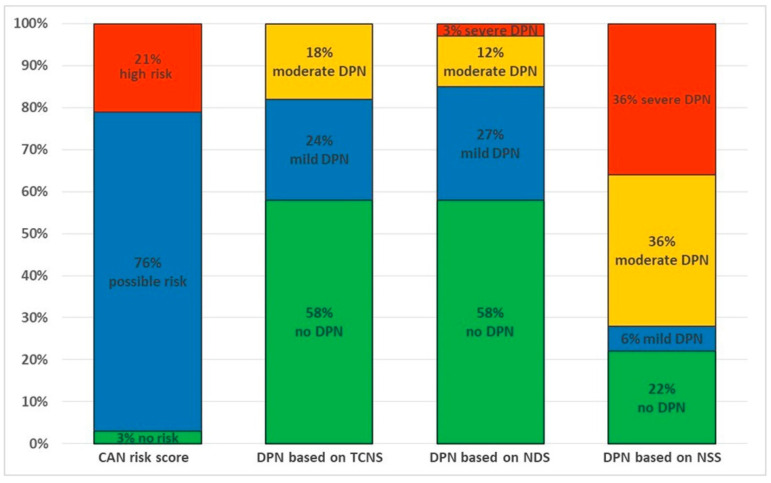
CAN risk score estimated by SUDOSCAN and DPN based on TCNS, NDS and NSS. Abbreviations: CAN—cardiac autonomic neuropathy, DPN—diabetic peripheral neuropathy, TCNS—Toronto Clinical Neuropathy Score, NDS—Neuropathy Disability Score, NSS—Neuropathy Symptom Score.

Based on the risk of CAN (SUDOSCAN CAN risk score) estimated by SUDOSCAN, 25 (75.8%) patients were identified at possible risk for CAN, while 7 (21.2%) were found to be at high risk (Figure 2). 

Based on the TCNS, neuropathy was present in 14 (42.4%) patients. Of these, eight were mild (24.2%) and six were moderate (18.2%). No patient experienced severe neuropathy based on the TCNS (Figure 2). 

According to the NDS score, neuropathy was present in 14 (42.4%) patients, where 9 were mild (27.3%), 4 were moderate (12.1%) and 1 was severe (3%) (Figure 2).

Based on the NSS score, neuropathy was found to be present in 26 (78.8%) patients, and where 2 were mild (6.1%), 12 were moderate (36.4%) and 12 were severe (36.4%) (Figure 2). 

The relationship between CAN risk and the ESC values, SBP, DBP, the level of the glycated hemoglobin, as well as the TCNS, NDS and NSS was examined using Pearson correlation coefficients.

**Figure 3:**
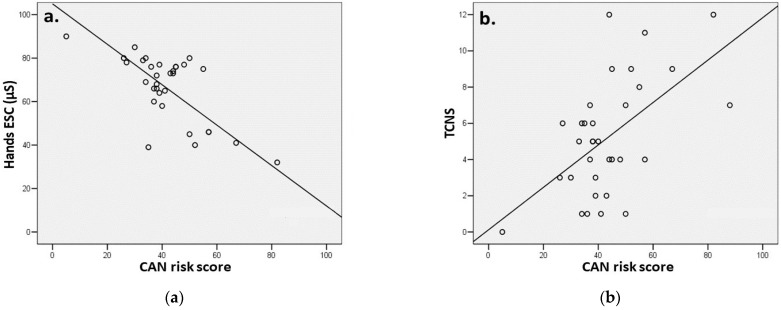
Correlations between the SUDOSCAN CAN risk, hand ESC (**a**) and TCNS (**b**). Abbreviations: CAN—cardiac autonomic neuropathy, ESC—electrochemical sweat conductance, TCNS—Toronto Clinical Neuropathy Score.

The SUDOSCAN CAN risk score was positively correlated with the hand ESC (r = 0.77, *p* < 0.001), the DBP (r = 0.35, *p* = 0.048), the level of the glycated hemoglobin (r = 0.46, *p* = 0.007), the TCNS (r = 0.56, *p* = 0.001), the NDS (r = 0.59, *p* < 0.001) and NSS (r = 0.37, *p* = 0.037) (Figure 3). 

There were no significant associations between the SUDOSCAN CAN risk score and other factors such as age, diabetes duration or SBP, with the correlation closest to positive values being between the BMI and the risk score, but still slightly in the negative field.

We also compared the group of patients at no (or low) risk for CAN (SUDOSCAN CAN risk score < 50%), with the one composed of patients found to be at high risk (SUDOSCAN CAN risk score ≥ 50%). Hand ESC was significantly lower in patients at high risk for CAN (*p* = 0.002). Moreover, the absence of reflexes (knee and ankle) was significantly higher with diabetic patients found to be at high risk for CAN (*p* = 0.008 and *p* = 0.029, respectively), followed by the absence of temperature and vibration sensation, as well as by the presence of weakness. Even if the DBP and the level of the glycated haemoglobin were higher in patients at high risk, no significant statistic associations were observed (Table 2). 

**Figure 4:**
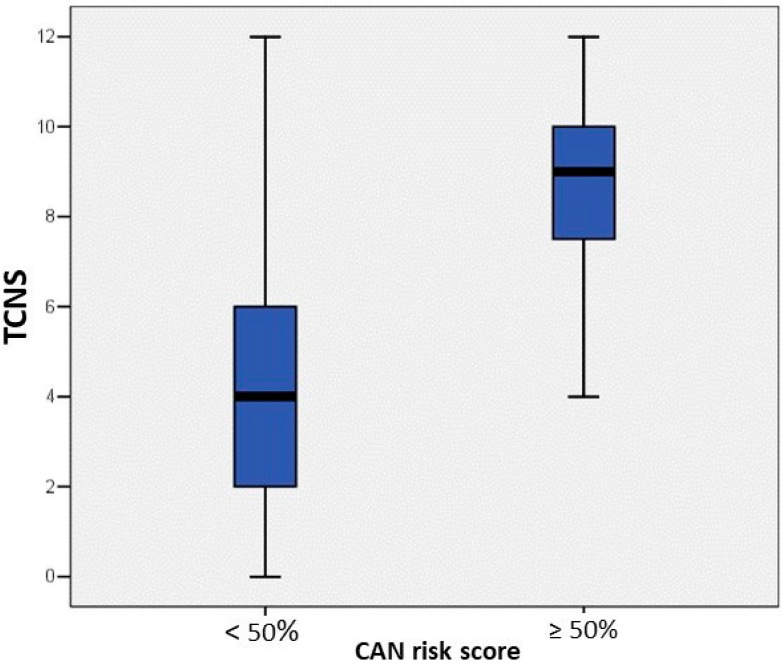
TCNS in patients found at high and low or no risk for CAN. Abbreviations: CAN—cardiac autonomic neuropathy, TCNS—Toronto Clinical Neuropathy Score.

TCNS was significantly higher in the case of patients found at high risk, compared to those at low or no risk for CAN (*p* = 0.001) (Figure 4). 

In the multiple linear regression analysis (SUDOSCAN CAN risk-score-dependent variable; diabetes duration, SBP, DBP and TCNS-independent variables), the SUDOSCAN CAN risk score was independently associated with higher DBP and higher TCNS (Table 3).

## 4. Discussion

CAN is a serious but mostly underdiagnosed complication of diabetes mellitus, with significant clinical and prognostic relevance [12]. It is also related to an increased risk of cardiovascular mortality from diabetes [26]. Most of the time, clinical symptoms of autonomic dysfunction only appear late after the onset of diabetes, and the global authorities have not come together on a common single approach for the diagnosis of CAN in diabetic patients [11].

The SUDOSCAN medical device was recently developed to detect early diabetic autonomic neuropathy and screen for CAN through the assessment of sudomotor function. The SUDOSCAN CAN risk score is derived from ESC, age, height, weight and the level of the glycated hemoglobin and was reported to have significant associations with methods generally used for the investigation of CAN, such as heart rate variability testing and Ewing tests [26]. However, in general, these tests are considered to be tough to perform, and thus, are rarely carried out in daily practice [18]. Unlike the traditional methods of CAN testing, the SUDOSCAN test is simple to perform, painless and non-invasive. It requires little staff technical training, no calculations, no subject preparation and, in addition to conventional tests, may be used for the early screening of CAN in everyday clinical practice, as robust results are obtained within minutes.

In the present study, CAN was assessed by measuring ESC through the SUDOSCAN medical device. ESC reflects sudomotor function, and both sympathetic and parasympathetic innervation contribute to normal sweat gland function [23,27]. Various studies have demonstrated that the method could represent a useful and simple tool for the early identification of DN, as well as in the screening of autonomic dysfunctions, including sub-clinical CAN [1,13,19,20,22,28].

Diabetic neuropathy intrinsically affects the longest fibers, such as those of the parasympathetic system. It is well known that cardiac alterations initially start with a relative increase in sympathetic tone. Denervation begins by affecting the heart at the apex, gradually impairing ventricular function and resulting in cardiomyopathy [10].

As previously presented in our study, the CAN risk score assessed by SUDOSCAN was positively correlated with hypertension, especially with the DBP. Even if the association between CAN and hypertension has not been extensively studied in diabetic patients so far, positive correlations between autonomic dysfunction and hypertension have also been observed in other studies. A variety of hypotheses concerning the causal relationship between CAN, hypertension and diabetes have been described [26,29,30]. Hypertension is common in patients with diabetes, and several data stand in favor of a role of enhanced sympathetic activity in the increase in blood pressure [31,32]. Sympathetic factors may also contribute to arterial stiffness. In this context, cardiac autonomic dysfunction estimated by sudomotor function has been recently correlated with arterial stiffness, even independently of conventional factors and glucose tolerance status [27]. 

We also observed that more patients at no or low risk for CAN received antihypertensive treatment, compared with those that had been found to be at high risk. Although at the moment there is not a pharmacological treatment for CAN, the nocturnal administration of antihypertensive drugs may ameliorate the 24 h blood pressure profile [33].

The SUDOSCAN CAN risk score has also been positively correlated with the level of glycated hemoglobin. Hyperglycemia is considered to play an essential role in the activation of several biochemical pathways linked to the metabolic and/or redox status, which, with impaired nerve perfusion, contribute to the development and progression of diabetic neuropathies [20]. Nevertheless, in diabetes, CAN, in the end, is the outcome of complex interactions between a degree of glycemic control, the duration of the disease, age-related neuronal attrition and systolic and diastolic blood pressure [34,35].

An important finding of the study was that SUDOSCAN CAN risk score was also correlated with the TCNS, NDS and NSS, thus indicating an association between CAN and peripheral neuropathy. The TCNS has been preferred in some clinical trials owing to its ease of use, acceptability by patients, its ability to classify the severity of diabetic sensorimotor polyneuropathy (DSP) and its representation of the clinical changes associated with the progression of DSP [36,37]. TCNS, therefore, has sufficient reliability and reproducibility as an instrument to document and monitor DSP in the clinical setting [36].

Currently, the gold standard in the evaluation of microfibers is considered to be a skin biopsy with intraepidermal nerve fiber density (IENFD). However, lately, sudorimetric techniques have undergone dramatic evolution, with ESC showing promising potential for disease detection, progression and response to therapy [23]. QSART, the most commonly used test of sudomotor function, has a high diagnostic sensitivity for small-fiber neuropathy. However, for optimum reliability, QSART requires meticulous attention to detail, including the maintenance of a constant environmental temperature. Maximal QSART reliability is achieved with an overnight fast and by holding medication for 48 h prior to the test, which in clinical practice is frequently impossible due to the risk of medical complications or symptom exacerbation with abrupt medication cessation, particularly in a diabetic population [13]. The measurement of ESC is an attractive alternative to skin biopsy and QSART. It is easy and rapid to perform and very well tolerated.

The limitations of the study include a relatively small sample size and the lack of other tests for CAN (such as the ones usually based on heart rate variability or Ewing tests), which are considered to be the gold standard for cardiac autonomic function and could be used to confirm our findings.

However, this was the first study performed in Romania using SUDOSCAN as a novel test for markers of CAN, a country in which little is known about the prevalence of CAN in diabetic patients. Therefore, the use of SUDOSCAN can be considered as a promising approach for diabetic neuropathy screening in the middle-income Baltic–Balkan states.

## 5. Conclusions

A high prevalence of sudomotor dysfunction was observed in a group of diabetic patients with or without neuropathy symptoms. Based on the results of the study, SUDOSCAN, as a simple, rapid and non-invasive test applied in order to assess the sudomotor dysfunction, proved to be a reliable method for the early screening of CAN.

## Figures and Tables

**Table 1:** The demographic and clinical characteristics of the patients.

	N	Mean	Standard Error	Standard Deviation	Percentiles		
					25	50	75
Age	33	64.6	2.8	15.9	60.5	65.0	72.5
Diabetes duration		12.9	1.6	9.3	5.5	11.0	18.0
BMI		30.6	0.9	5.4	26.2	30.0	34.2
SBP		149.6	4.0	23.1	130.0	150.0	162.5
DBP		82.7	1.7	9.7	77.5	80.0	90.0
Glycated hemoglobin		7.4	0.3	1.5	6.3	7.2	8.7

N—number of patients; BMI—body mass index; SBP—systolic blood pressure; DBP—diastolic blood pressure.

**Table 2:** Comparison of different parameters between the group of patients at no (or low) risk for CAN (SUDOSCAN CAN risk score < 50%) with the one composed of patients found to be at high risk (SUDOSCAN CAN risk score ≥ 50%).

Parameter	*SUDOSCAN* CAN Score—Risk		*p*
	Risk Score ≤ 50 (*N* = 26)	Risk Score > 50 (*N* = 7)	
	Mean ± Standard Deviation	Mean ± Standard Deviation	
Men *n* (%)	13 (50.0)	5 (71.4)	0.413
Age (years)	63.5 ± 17.0	68.9 ± 10.8	0.643
Diabetes duration (years)	12.6 ± 8.7	13.9 ± 11.8	0.965
BMI (kg/m^2^)	30.7 ± 5.5	30.3 ± 5.6	0.889
Glycated hemoglobin (%)	7.2 ± 1.2	8.5 ± 2.0	0.094
SBP (mmHg)	148.3 ± 25.1	154.3 ± 14.0	0.549
DBP (mmHg)	81.6 ± 10.4	87.1 ± 4.9	0.161
Hand ESC (µS)	71.0 ± 11.3	40.6 ± 21.0	0.002
Feet ESC (µS)	67.4 ± 17.9	43.0 ± 29.6	0.058
Antihypertensive treatment (%)	23.0 (88.5)	3.0 (42.9)	0.023
Smoking (%)	3.0 (11.5)	1.0 (14.3)	0.639
Retinopathy (%)	7.0 (26.9)	3.0 (42.9)	0.646
Arteriopathy (%)	6.0 (23.1)	2.0 (28.6)	1.000
Nephropathy (%)	2.0 (7.7)	0.0 (0.0)	1.000
TCNS	4.3 ± 2.7	8.6 ± 2.6	0.001
NDS	1.9 ± 1.9	6.0 ± 2.6	0.001
NSS	4.7 ± 2.9	6.7 ± 2.1	0.237

N—number of patients; BMI—body mass index; SBP—systolic blood pressure; DBP—diastolic blood pressure; ESC—electrochemical sweat conductance; TCNS—Toronto Clinical Neuropathy Score; NDS—Neuropathy Disability Score; NSS—Neuropathy Symptom Score.

**Table 3:** Multiple linear regression analysis (SUDOSCAN CAN risk-score-dependent variable; diabetes duration, SBP, DBP and TCNS-independent variables).

Parameter	Non-Standardized Coefficients		Standardized Coefficients	*p*	Confidence Interval (95%)	
	B	Standard Error	Beta			
Constant	11.1	19.9	-	0.580	−29.6	51.9
Diabetes duration (years)	0.3	0.2	0.2	0.304	−0.3	0.8
SBP (mmHg)	−0.2	0.1	−0.3	0.073	−0.5	0.0
DBP (mmHg)	0.6	0.3	0.4	0.046	0.0	1.2
TCNS	2.3	0.8	0.5	0.005	0.8	3.8

SBP—systolic blood pressure; DBP—diastolic blood pressure; TCNS—Toronto Clinical Neuropathy Score.

## Data Availability

The data presented in this study are available on request from the corresponding author. The data are not publicly available due to privacy matters.
